# Classifying tasks performed by electrical line workers using a wrist-worn sensor: A data analytic approach

**DOI:** 10.1371/journal.pone.0261765

**Published:** 2022-12-09

**Authors:** Saeb Ragani Lamooki, Sahand Hajifar, Jacqueline Hannan, Hongyue Sun, Fadel Megahed, Lora Cavuoto

**Affiliations:** 1 Mechanical and Aerospace Engineering, University at Buffalo, Buffalo, NY, United States of America; 2 Industrial and Systems Engineering, University at Buffalo, Buffalo, NY, United States of America; 3 Biomedical Engineering, University at Buffalo, Buffalo, NY, United States of America; 4 Farmer School of Business, Miami University, Oxford, OH, United States of America; Universiti Tunku Abdul Rahman, MALAYSIA

## Abstract

Electrical line workers (ELWs) experience harsh environments, characterized by long shifts, remote operations, and potentially risky tasks. Wearables present an opportunity for unobtrusive monitoring of productivity and safety. A prerequisite to monitoring is the automated identification of the tasks being performed. Human activity recognition has been widely used for classification for activities of daily living. However, the literature is limited for electrical line maintenance/repair tasks due to task variety and complexity. We investigated how features can be engineered from a single wrist-worn accelerometer for the purpose of classifying ELW tasks. Specifically, three classifiers were investigated across three feature sets (time, frequency, and time-frequency) and two window lengths (4 and 10 seconds) to identify ten common ELW tasks. Based on data from 37 participants in a lab environment, two application scenarios were evaluated: (a) intra-subject, where individualized models were trained and deployed for each worker; and (b) inter-subject, where data was pooled to train a general model that can be deployed for new workers. Accuracies ≥ 93% were achieved for both scenarios, and increased to ≥96% with 10-second windows. Overall and class-specific feature importance were computed, and the impact of those features on the obtained predictions were explained. This work will contribute to the future risk mitigation of ELWs using wearables.

## 1 Introduction

The reliable delivery of energy is a crucial factor of economic development and a key metric to assess a developed nation’s infrastructure [1]. Electric power reliability demands a high level of maintenance, which is performed by Electric Power Industry Workers (EPIWs). Among the EPIW, Electrical Line Workers (ELWs) have the third highest injury rate after meter readers and welders [2]. For an overall injury rate of 3.2 per 100 employee-years for the EPIWs from 1995 to 2013, the ELWs injury rate was 10.37 based on the data collected from 18 electric power companies in the US [3]. Among the tasks that ELWs perform, walking up and down ladders and stairs; climbing down poles and transmission towers; entering, stepping out of, or approaching the utility trucks, bucket, or vaults; and performing repetitive work/overtime are found to be associated with the highest risks of injury [4]. Thus, timely monitoring and analysis of ELWs activities can provide significant information about the type, duration, and frequency of risky tasks and can be used for prevention of work-related injuries [5].

**Fig 1:**
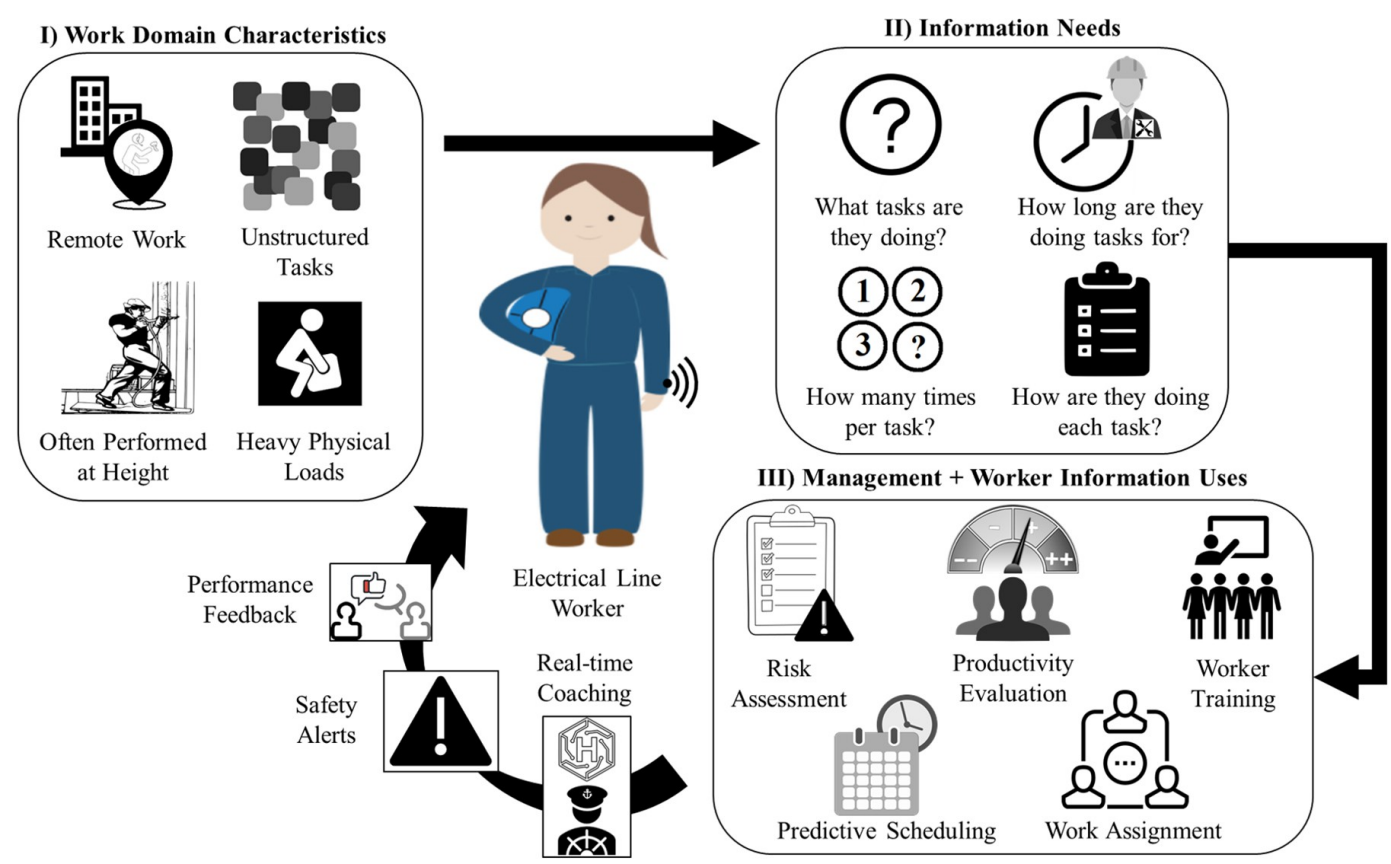
Use of IoT for safety enhancement.

In the past decade, the Internet of Things (IoT) has enabled the collection of data to elucidate system operations and facilitate smart operation decisions to enhance several key performance measures [6–8]. From a safety perspective, wearable sensors and IoT-based monitoring can be used to estimate the risk of injury and support risk reduction [9–13]. In addition to risk assessment, IoT data analysis can support worker training, work assignment, and predictive scheduling, and the outcomes can ultimately be fed back to the worker in the form of feedback and safety alerts. In the current application, we are interested in leveraging IoT to collect kinematic data from ELWs. As shown in Fig 1, there are work domain characteristics for electrical work that support the use of IoT for safety enhancement and that can be captured through kinematic analysis, including the nature of the work tasks (e.g., often performed at height and with heavy loads), variability in tasks across days, and risk in remote/lone worker situations. Worker kinematics allows for monitoring which tasks are performed, how many times, and for what duration, all of which are critical factors for risk assessment.

With the kinematics data, one needs to first identify the activities that ELWs performed, to track the time and duration of these activities and to guarantee the worker safety and prevent economic loss. Therefore, a robust activity recognition method that can be effectively integrated to the ELWs work settings for detection of relevant activities is of interest. In the literature, many studies have investigated the application of human activity recognition (HAR) methods in activities of daily living (ADL), sports, and healthcare [14–16]. However, the activities involved in industrial occupations are often more complex and the complexity further varies between different industries. The application of HAR methods in occupational activities has received attention in certain industries, such as construction [5, 17, 18] and assembly line works [19–21]. Considering the uniqueness of the tasks in different industries, the effectiveness of HAR algorithms needs to be investigated independently in each industry. Particularly, we are interested in classifying common tasks among ELWs, such as climbing ladders, hoisting tools, and working on electrical panels. Moreover, in HAR applications different hardware and software challenges need to be addressed based on the limitations in the industry. For ELWs due to the long shifts in remote locations, the hardware must be lightweight and able to operate long hours on a single charge, while preserving a high recognition accuracy. Towards this goal, we investigated the feasibility of a single wrist-worn accelerometer for activity recognition among ELWs.

In HAR applications an inherent trade-off exists between the number and location of the sensors/signals and the feasibility of the method. The accuracy of the classification methods generally increases with more sensors/signals at different body locations. However, this can disrupt the underlying task in real-world settings. In occupational applications, it is critical to attach the sensor set to the worker with minimum obtrusiveness to allow maximum flexibility for complex motions [21]. The performance of HAR algorithms depends on the location of the sensors on the body, since the acceleration signals vary at different body locations while performing the same activity [22]. Since in many occupational tasks the upper limb motions and particularly the hand kinematics contain significant information about the underlying activities [22], we have investigated the effectiveness of HAR methods to classify the ELWs activities using a single accelerometer on the dominant wrist.

The application of individual sensors on the upper limbs for activity recognition has been investigated under certain circumstances. Nath et al. [5] attached a single smartphone to the upper arm and reported the highest accuracy of 90.2% for a 3-class classification of construction tasks. They used three measurements from the smart phone’s built-in accelerometer, linear accelerometer, and gyroscope. Koskimaki et al. [20] used an inertial measurement unit (IMU) on the dominant wrist to classify 5 activities of assembly line workers in automotive industry using the accelerometer and gyroscope data and reported 90% accuracy. Using a single wrist-worn accelerometer, Ryu et al. [18] classified 4 construction activities and reported the best accuracy of 88.1%. Also, Yang et al. [23] utilized a single accelerometer on the dominant wrist to classify 8 domestic activities and reported 95% accuracy. Additionally, by the use of a single wrist-worn accelerometer, Chernbumroong [24] classified 5 ADLs and reported the best accuracy of 94.13%. Classification of occupational tasks are in general more challenging than the ADLs due to the complexity of the motions and therefore lower accuracy is reported for them in the literature especially when fewer sensors/signals are used. We have summarized the activities, classification methods, number of participants, sensor type and location, and the reported results from a number of studies that used a single sensor on the upper limbs in Table 1.

**Table 1:** Activity recognition studies using a single sensor worn on upper limb.

Reference	Activities	Classification Method	#Subjects	Sensors and Location	Highest Reported Result
[23]	8 ADLs: Standing, Sitting, Walking, Running, Vacuuming, Scrubbing, Brushing Teeth, and Working at a Computer	Neural Network	7	Accelerometer; Wrist	Accuracy = 95%
[20]	5 Tasks: Hammering, Screwing, Spanner Use, Using Power Drill for Screwing, and Null Activity	*k*-NN	4	Accelerometer & Gyroscope; Wrist	Accuracy = 90%
[25]	11 Tasks: 10 Upper Body Exercises (Butterflies, Chest Press, Latissimus, Abdominal, Upper Back, Shoulder Press, Pulldown, Low Row, Arm Curl, Arm Extension) mixed with a Null class	*k*-NN	7	Accelerometer and Gyroscope; Upper Arm	Accuracy = 93.6%
[24]	5 ADLs: Lying, Sitting, Standing, Walking, Running	Decision Tree, Artificial Neural Network	7	Accelerometer; Wrist	Accuracy = 94.13%
[26]	Case 1. 10 Tasks: Lying, Standing, Walking, Sitting, Cycling, On All Fours, Kneeling, Running, Bending, and Transition, Case 2. 4 Tasks: Standing, Walking, Sitting and Lying	Deep Learning Convolutional Network, Random Forest with Hand Crafted Features	Case 1: 10, and Case 2: 03	Accelerometer; Wrist Case 1.	Accuracy = 74.6%, Case 2. 66.8%
[27]	5 Tasks: Loading Sections into Wheelbarrow, Pushing a Loaded Wheelbarrow, Dumping Sections from Wheelbarrow, Returning an Empty Wheelbarrow, and Being Idle	Neural Network, Decision Tree, *k*-NN, Logistic Regression and SVM	2	Accelerometer and Gyroscope; Upper Arm	Accuracy = 80% (for intersubject case and category 3)^1^
[5]	3: 2 High Risk Categories as lifting/ lowering/carrying (category-1), and pushing/pulling (category-2) and 1 Low Risk Category	SVM	2	Accelerometer, Linear Accelerometer, and Gyroscope; Upper Arm	Accuracy = 90.2%
[28]	6 Tasks: Grab Tool/Part, Hammer Nail, Use Power-screwdriver, Rest Arm, Turn Screwdriver and Use Wrench	Convolutional Neural Network	8	Accelerometer, Angular Velocitimeter, 4-channel Orientation and sEMG; Forearm	Accuracy = 98%
[18]	4 Tasks: Spreading Mortar, Laying Blocks, Adjusting Blocks, and Removing Mortar	*k*-NN, Multilayer Perceptron, Decision Tree and Multiclass Support Vector Machine	10	Accelerometer; Wrist	Accuracy = 88.1%
[29]	Case 1. 13 Tasks: Four gross motion dashboard assembly tasks, and nine fine motion block assembly and grasping tasks, Case 2. 30 strenuous gym exercises, representative of lifting, pushing, and carrying tasks	CNN, LSTM, CNN-LSTM, *k*-NN and LDA	Case 1: 5, Case 2: 10	Accelerometer, Gyroscope and sEMG; Forearm	Case 1. F1 Score = 36%, Case 2. F1 Score = 84%

^1^ Here, we report their third category which has more classes compared to their other categories.

**Fig 2:**
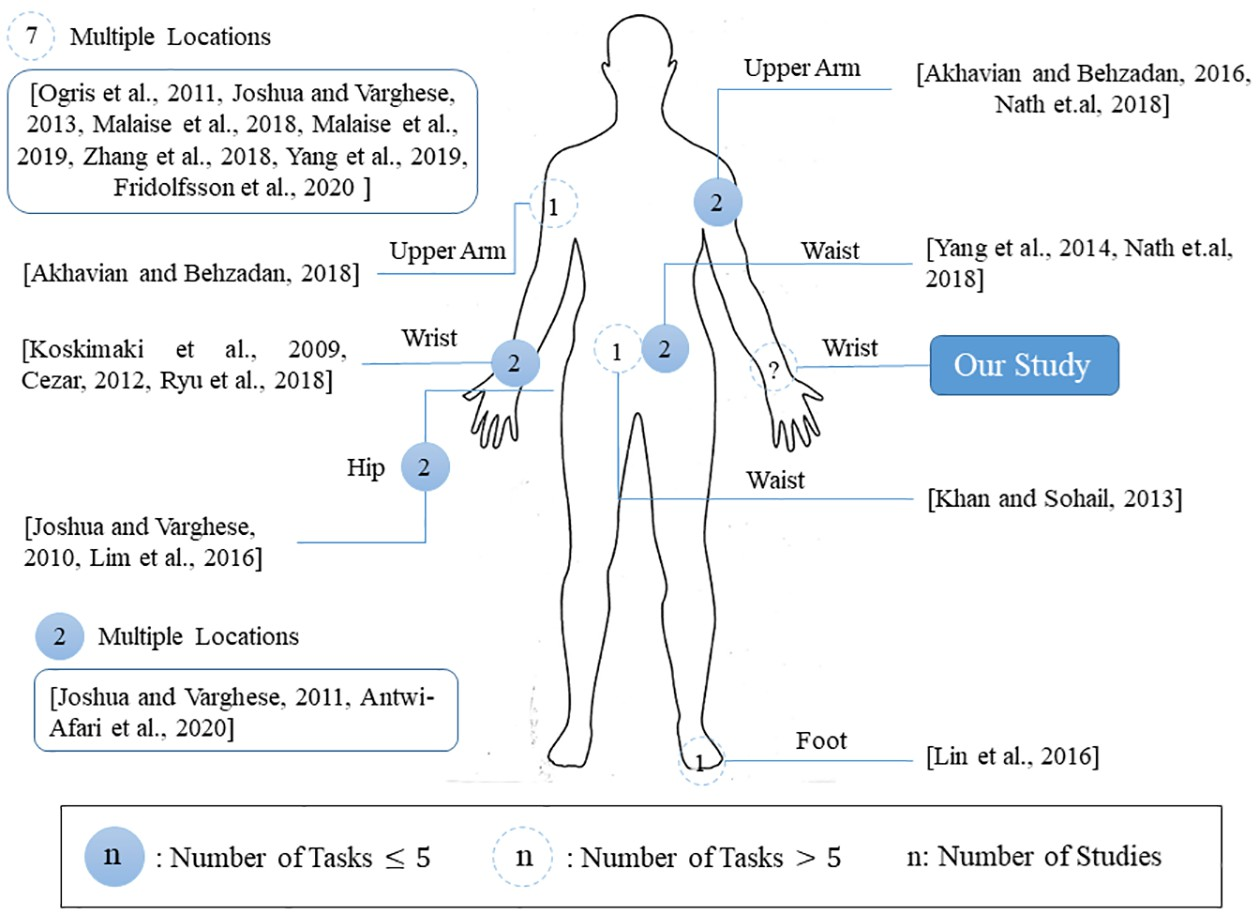
Body locations of the wearable sensors across the reviewed studies that involved occupational tasks (20 studies).

Choosing sensor attachment location(s) is a crucial decision when using wearable sensors [30], which depends on the application and types of the tasks. Here, we review the sensor attachment locations and number of the activities in related occupational studies as depicted in Fig 2. The number of sensors utilized per study ranges from 1 to 24, with a median of 1. Fig 2 illustrates the total number of times a wearable sensor was placed at a certain body location (shown by circles), with 71 sensor attachments across the 20 studies surveyed. Those studies with multiple sensor placements are indicated in the upper left and lower left of Fig 2. In 40 out of 71 sensor placements, the sensors were placed bilaterally (∼ 56%). In case a sensor was placed unilaterally, placement on either the dominant side or the right side was more common (18 out of 20) compared to non-dominant side or left side (2 out of 20). The wrist was the most often used location for sensor placement (6 out of 20 studies), followed by waist, upper arm and foot (5 of 20 studies each).

Moreover, we categorize the studies into two groups based on the number of tasks/classes and present it in Fig 2 where fewer than 6 tasks are shown by blue circles and 6 or more tasks are shown by white circles. The number of tasks per study ranged from 3 to 23, with a median of 5.5. The studies that included more than 5 tasks used multiple sensors at multiple locations more often (7 out of 9). For instance, [31] used four wrist-mounted sensors to classify 23 bicycle maintenance tasks and, sometimes, the classification involved distinguishing between the tasks as similar as fastening and unfastening a screw (both of them require rotational motion in both directions). On the other hand, the studies considering fewer tasks were more likely to use a single sensor (8 out of 10). However, [32, 33] tried to classify large number of classes using a single wearable sensor to reduce the obtrusiveness. From Fig 2, it is clear that using a single wrist-mounted sensor to classify large number of classes was not explored across the 20 studies that investigated the occupational tasks, which is the focus of our research [5, 18, 20, 24, 27, 31–46]. Besides being less obtrusive, wristband sensors are commercially available and easily adoptable by the consumers [47, 48].

Based on a single wrist-worn sensor, this paper aims at proposing a data-driven methodology for accurate activity recognition for ELWs. To achieve this goal, we have addressed three research questions: (1) What is the predictive performance obtained from using a single wearable sensor to classify different ELWs tasks? (2) How well does the approach in research question 1 generalize to individuals not used in the training of the models? and (3) What is the relative importance and impact of the features extracted from the wearable sensor in order to distinguish the performed tasks?

To address these questions, we will achieve automated human activity recognition for ELWs, capitalizing on existing feature extraction and statistical learning techniques. Specifically, we designed an in-lab experiment to examine:

1. *Intra-subject Classification*: We perform the activity recognition for 10 ELWs’ tasks using *k*-nearest neighbors (*k*-NN), support vector machine (SVM), and random forest (RF) models with the data from a wearable sensor for each subject.
2. *Inter-subject Classification*: We further test the extrapolation performance of the activity recognition for new participants, to determine whether our models are robust to the heterogeneity across participants.
3. *Interpretation of Predictions*: In addition to the overall feature importance, we analyze the task-specific feature importance, since the contribution of different features varies for prediction of different classes, using local interpretable model-agnostic explantions (LIME) method [49].

Addressing these questions paves the way for automatic and remote monitoring of the duration and frequency of the hazardous tasks performed by ELWs.

## 2 Methods

### 2.1 Experiment design and data collection

We recruited 37 participants (demographic and anthropometric data summarized in Table 2) to perform experimental sessions that included 10 simulated common activities of ELWs. The study was approved by the University at Buffalo Institutional Review Board and all participants provided signed informed consent prior to participating. At the start of the session, participants were instrumented with an Empatica E4 wristband (Empatica, Boston, United States) on their dominant wrist to collect the acceleration in the *X*, *Y*, and *Z* directions (with respect to the local coordinates of the accelerometer) at a sampling rate of 32 Hz. Participants then completed the series of 10 activities as presented in Table 3. The participants were asked to carry a smartphone in their pocket for real-time collection of data from the wristband via the E4 real-time app and Bluetooth connection. The data were uploaded to the cloud automatically by the app at the end of the data collection session. The start and end time of each activity was recorded by an annotator during the session.

**Table 2:** Summary of anthropometric data for the participants. The values in columns 3–5 are the average (± standard deviation).

Gender	Count	Age	Body Mass (kg)	Height (m)
Male	23	27.61 (± 4.16)	80.07 (± 15.33)	1.80 (± 0.08)
Female	14	23.36 (± 5.14)	63.29 (± 14.48)	1.63 (± 0.07)

**Table 3:** The experimental tasks, which are performed in a columnwise order by row, i.e., first column → second column → third column.

General Mobility (Duration)	General Work Tasks (Repetitions)	Specialized Work Tasks (Duration)
Sit (3 min)	Hoist (10 rep, 3.5 kg per rep)	Type on a Computer (3 min)
Stand (3 min)	Lift & Lower a Box (20 rep, 10 kg per box)	Work at Electrical Panel (3 min)
Walk (3 min)	Push a Cart (10 rep, 10 kg load)Ascend & Descend a Ladder (20 rep)	Overhead Screw (3 min)

### 2.2 Workflow

**Fig 3:**
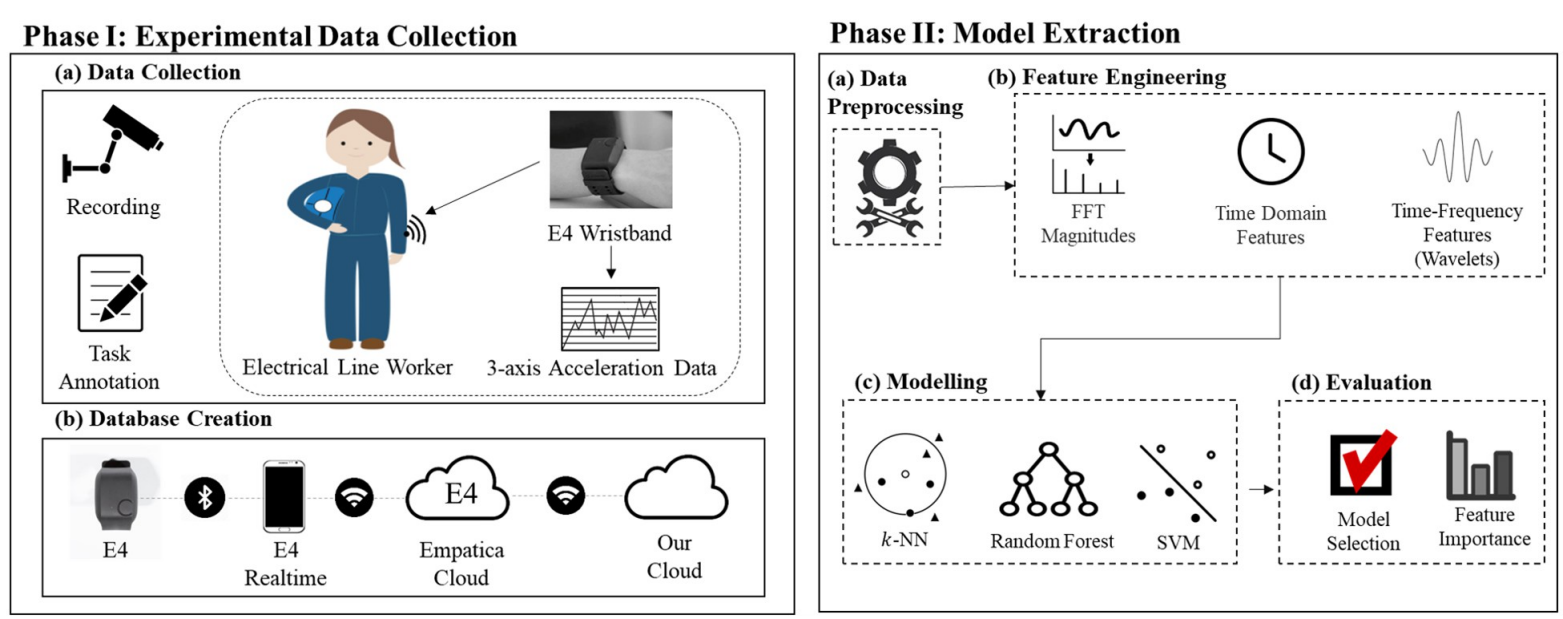
An overview of activity recognition framework for acquiring, preprocessing, and analyzing acceleration data.

Our analysis workflow is presented in Fig 3. After the data was collected and stored locally, the tasks were annotated and the signals within each task were segmented to windows. Next, three types of features in the time, frequency, and time-frequency domains were generated within each window. The features were used as the inputs to three classifiers: *k*-NN, SVM, and RF. The classifiers were trained and tested for intra-subject and inter-subject scenarios. The best set of window length, feature set, and classifier were selected based on the inter-subject analysis for potential deployment.

### 2.3 Windowing

**Fig 4:**
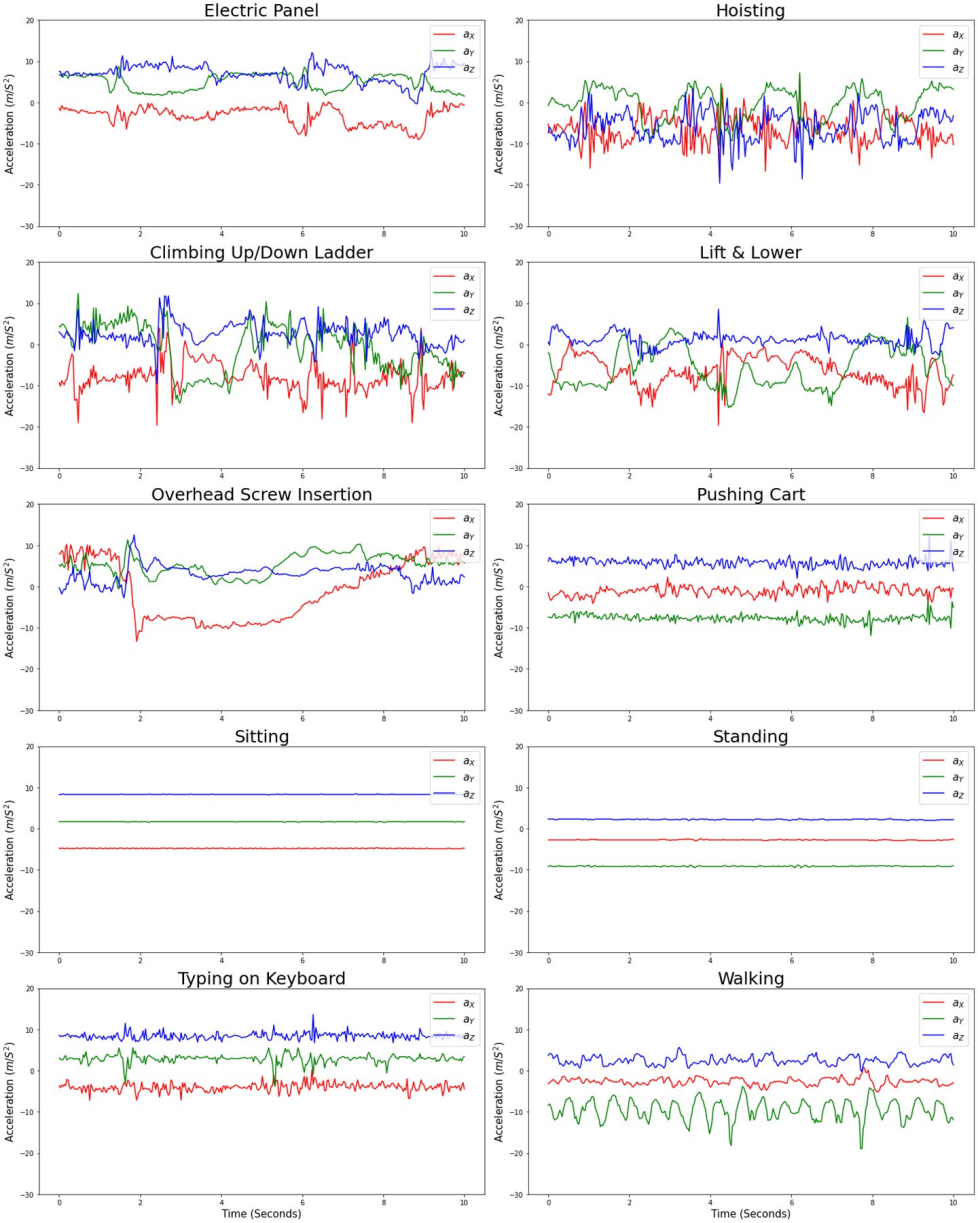
Acceleration signals in *X*, *Y*, and *Z* directions in a 10 seconds window presenting the 10 activities for a sample subject.

We labeled the acceleration signals and segmented the annotated signals into equal length windows. Multiple windowing strategies have been used in the HAR literature, including non-overlapping sliding windows, overlapping sliding windows, event-defined windows, and activity-defined windows [50]. In our work, we segmented the labeled acceleration data into non-overlapping fixed-sized windows, as this is the most widely employed windowing technique, due to simplicity and efficient pre-processing [51]. Considering the complexity of the activities and the use of a single accelerometer, we selected two different window lengths of 4 and 10 seconds, equivalent to 128 and 320 data points in the acceleration signal (sampling rate of 32 Hz). While smaller window sizes can result in faster activity recognition, larger windows are better suited for recognition of complex activities. Banos et al. reported the optimal window size of 0.5 to 6.75 seconds in sport/wellness applications [51]. In Fig 4 we visualized the three acceleration signals in *X*, *Y*, and *Z* directions without any filtering within a segmented window of 10 seconds for the 10 activities of a single participant.

### 2.4 Feature extraction

We have focused on using the shallow features in our study since the deep features from deep learning algorithms require larger data sets to train, and are difficult to interpret, due to their black box nature [52, 53]. In this study we examined the performance of features from 3 domains: time, frequency, and time-frequency. Time domain features include information regarding the central tendency, average power, dispersion, and the range of the signal. The frequency domain features capture the central tendency and dispersion of the signal by the FFT coefficients at lower and higher frequencies, respectively. While the frequency domain features include more detailed information about the frequency components of the signal as compared to the time domain features, they are better suited for stationary signals. Given the complexity of certain tasks in our data set, windows / observations with non-stationary signals are present in our data. Therefore, we also evaluated the performance of time-frequency (wavelet) domain features as they perform better for non-stationary signals.

Time domain features are commonly statistical measures that are simple to compute [54, 55]. However, these features are unable to describe the signal’s functional shape [56], and in spite of their good performance for distinguishing between the static and dynamic tasks, they often do not perform well in differentiating between dynamic activities [55]. To extract the time domain features in our study, we first removed the high frequency noise by applying a 4^*th*^ order low pass Butterworth filter with a cutoff frequency of 10 Hz on the three acceleration signals within each window. The cut-off frequency of 10 Hz was selected since the acceleration signals associated with the human movements usually do not exceed 10 Hz [57, 58]. Next, we calculated 9 statistics for each of the three filtered signals within the window. These statistics included mean to capture the central tendency of the signal, root mean square (RMS) to represent the average power of the signal, mean average deviation (MAD) and standard deviation (*σ*_*x*_) to represent the signal dispersion, and minimum, maximum, median, 25^*th*^ percentile, and 75^*th*^ percentile as the summary statistics of the signal. The 9 statistics per signal summed up to a total of 27 time domain features for each window/observation.

The frequency domain features provide information about the intensity of different frequency components in the raw signal. Advantages of frequency domain features are robustness to noise and interpretability [58, 59]. On the other hand, since the frequency domain features do not provide information about the timing of frequency components [60], their performance is limited in the case of non-stationary signals [61]. We transformed the three acceleration signals to the frequency domain using fast Fourier transform (FFT). To remove the data associated with high frequency noise, we only preserved the FFT coefficients at up to 10 Hz. This resulted in feature vectors of length 120 and 300 for the 4 and 10 second windows, respectively.

The time-frequency domain features preserve the frequency information at each time point. We used discrete wavelet transform (DWT) for extraction of the time-frequency features. The DWT features are more reliable for modelling non-stationary acceleration signals [62]. Moreover, the DWT features can represent sharp peaks and abrupt changes [63], enabling the detection of transitions between postures [64]. However, compared to FFT coefficients, the DWT coefficients are less interpretable. For the DWT transformation, we used a Daubechies-4 mother wavelet (db 4) with the filter bank approach to decompose the original signals to approximation (cA) and detail (cD) coefficients. The decomposition was applied up to the maximum possible level on the three acceleration signals (4 and 5 levels for the cases of 4 and 10 second windows, respectively). To reduce the dimension of the wavelet coefficeints, entropy, number of zero- and mean-crossings, and the 9 statistics used for time features were calculated for the last approximation coefficients and all detail coefficients and were concatenated to generate the time-frequency features. This resulted in features with the lengths of 180 and 216 for the 4 and 10 second windows, respectively.

The utility of different feature sets depends on the classification application. Selecting the best set of features depends on the type of movements associated with different activities. It is noteworthy to mention that, even though using the acceleration magnitude instead of the 3 acceleration signals could decrease the number of features by two thirds and thereby decrease the computational cost, we included the 3 individual acceleration signals since they contain valuable information in terms of the accelerometer orientation and allow for more accurate classification. For instance, in Fig 4, it is evident that in sitting and standing tasks the gravity as the sole existing acceleration, appears as different components in the three Cartesian coordinates due to the different wrist orientation, whereas the acceleration magnitude is the same for the two tasks and cannot be used to distinguish them.

### 2.5 Classification and performance

We performed the activity recognition of the 10 activities in two scenarios of intra-subject and inter-subject settings. In each scenario, we investigated *k*-NN, SVM, and RF classifiers in combination with the two window lengths and the three feature sets discussed in the previous sections. We avoided adoption of deep learning since for the intra-subject analysis the number of observations are not enough for training such models. Moreover, the large computational cost and the lack of interpretability of deep learning models led to prioritization of conventional classifiers.

The training and testing splits have been performed differently for the intra- and inter-subjects cases. In both designs we tuned the classifier’s hyper-parameters using 10-fold cross validation and grid search. For the *k*-NN classifier, the grid search was performed for the number of neighbors (from 1 to 28 by increments of 3), the distance metric (Euclidean and Manhattan), and the weight (uniform and distance). For the SVM classifier the grid search was performed on the regularization parameter C (from 0.01 to 50), and the kernel (linear, polynomial, RBF, and Sigmoid). Also, for the RF classifier we performed the grid search on the maximum depth of the trees (5, 10, 20, 30, 40), and the number of features to consider for best split (20, 30, 40, 50, 60).

For the intra-subject classification we randomly split 80% of observations as training set and 20% of observations as testing set from the same subject. We used stratified sampling of each activity, to ensure balanced distribution of labels. Next, we used 10-fold cross-validation to select the optimum hyper-parameters as described above. We performed the hyper-parameter tuning for each participant independently and reported the mean testing accuracy, precision, recall, and F1 score across all participants.

In the inter-subject analysis, we split the subjects into five folds and repeated the training and testing five times, using each fold as the test data in one iteration and the remaining subjects as the training data. The best model hyper-parameters were chosen using 10-fold cross validation in the training data. We calculated the average overall accuracy of the test sets as well as the average precision, recall, and F1 score for individual activities in order to evaluate the classifier’s performance for different labels. In order to come to a conclusion regarding model performance, we performed a two-way analysis of variance (ANOVA) to evaluate the main and interactive effects of feature set and classifier on the overall accuracy. Post hoc comparisons for significant effects were performed using Tukey’s HSD. The significance level was set at *p* < 0.05.

### 2.6 Feature importance and interpretation

In this study, by finding the features that contributed the most to the predictions we attempted to investigate the mechanism based on which the classifiers performed. To that end, we obtained the overall feature importance from the RF classifier. In the case of multi-class classification, feature contribution differs by class, therefore, we further investigated the importance of features for prediction of individual classes. In the latter case, we used LIME to explain the predictions by perturbing the input data locally around a selected sample via an interpretable model such as a linear model [49]. Thus, we can better understand the feature importance during the prediction in each sample, and make the classification decisions more transparent.

## 3 Results

### 3.1 Intra-subject classification performance

Given the large number of participants, we present the average test accuracy, precision, recall, and F1 score over all participants in Table 4. The results are presented for all combinations of window length, feature set, and classifier. In the intra-subject scenario, the longer window length always resulted in better accuracy for any combination of the classifiers and feature sets. For the window length of 4 seconds, the time and time-frequency domain feature sets resulted in high accuracy, ranging between 94% and 96%, with SVM slightly outperforming the others. For the 10 second windows, the accuracy increased overall and the time and time-frequency domain features outperformed the frequency features. High accuracy of 99% was achieved for the *k*-NN and SVM classifiers with the time and time-frequency domain features.

**Table 4:** Intra-subject classification performance. Abbreviations: WL: window length, T: time, F: frequency EP: electrical panel, H: hoisting, Ld: ladder, Lf: lifting; OH: overhead; P: pushing; St: sitting; Sd: standing; Tp: typing; W: walking.

WL (s)	Feature	Model	Task-Specific Performance in the Test Set											Test Acc
				EP	H	Ld	Lf	OH	P	St	Sd	Tp	W	
4	T	*k*-NN	p	0.96	0.99	0.93	0.92	0.99	0.99	0.99	0.98	0.99	0.97	0.96
			r	1.00	0.95	0.94	0.94	0.97	0.99	0.98	0.94	0.96	0.98	
			f1	0.98	0.97	0.93	0.93	0.98	0.99	0.99	0.96	0.98	0.98	
4	T	SVM	p	0.96	0.98	0.92	0.94	0.99	0.99	0.99	0.94	0.99	0.97	0.96
			r	1.00	0.96	0.94	0.93	0.97	0.98	0.98	0.92	0.96	0.98	
			f1	0.98	0.97	0.93	0.93	0.98	0.98	0.98	0.93	0.98	0.98	
4	T	RF	p	0.96	0.96	0.89	0.89	1.00	0.98	0.99	0.97	0.99	0.95	0.95
			r	0.99	0.93	0.91	0.89	0.94	0.97	0.99	0.95	0.97	0.98	
			f1	0.97	0.94	0.90	0.89	0.97	0.97	0.99	0.96	0.98	0.97	
4	F	*k*-NN	p	0.84	0.92	0.76	0.71	0.94	0.88	0.98	0.93	0.96	1.00	0.86
			r	0.91	0.69	0.80	0.84	0.76	0.89	0.98	0.93	0.94	0.96	
			f1	0.86	0.78	0.78	0.76	0.83	0.88	0.97	0.92	0.95	0.98	
4	F	SVM	p	0.90	0.92	0.82	0.85	0.96	0.97	0.99	0.94	0.98	0.99	0.91
			r	0.98	0.87	0.87	0.84	0.89	0.94	0.98	0.94	0.94	0.96	
			f1	0.94	0.89	0.84	0.84	0.92	0.95	0.98	0.94	0.96	0.98	
4	F	RF	p	0.91	0.95	0.86	0.84	0.95	0.94	0.98	0.97	0.96	0.96	0.92
			r	0.94	0.83	0.89	0.90	0.84	0.96	0.97	0.94	0.93	0.98	
			f1	0.92	0.88	0.87	0.86	0.89	0.95	0.98	0.95	0.95	0.97	
4	T-F	*k*-NN	p	0.95	0.99	0.89	0.84	1.00	0.97	0.99	0.92	0.99	0.99	0.94
			r	0.99	0.91	0.88	0.91	0.95	0.97	0.98	0.93	0.95	0.98	
			f1	0.97	0.95	0.89	0.87	0.97	0.97	0.99	0.92	0.97	0.98	
4	T-F	SVM	p	0.96	0.99	0.86	0.89	1.00	0.98	0.98	0.95	0.99	0.99	0.94
			r	0.99	0.96	0.92	0.88	0.97	0.96	0.97	0.91	0.95	0.98	
			f1	0.97	0.97	0.89	0.88	0.98	0.97	0.97	0.92	0.97	0.99	
4	T-F	RF	p	0.94	0.98	0.90	0.92	1.00	0.98	1.00	0.96	0.99	0.98	0.95
			r	0.99	0.93	0.94	0.89	0.94	0.97	0.98	0.95	0.95	0.99	
			f1	0.96	0.95	0.91	0.90	0.97	0.97	0.99	0.96	0.97	0.98	
10	T	*k*-NN	p	0.99	1.00	0.99	0.99	1.00	1.00	0.99	1.00	0.99	1.00	0.99
			r	0.99	0.98	0.99	1.00	0.99	1.00	1.00	1.00	0.99	1.00	
			f1	0.99	0.99	0.99	0.99	1.00	1.00	1.00	1.00	0.99	1.00	
10	T	SVM	p	1.00	0.98	1.00	0.98	1.00	1.00	1.00	1.00	0.99	0.99	0.99
			r	1.00	1.00	0.98	0.99	0.99	1.00	0.99	0.99	1.00	1.00	
			f1	1.00	0.99	0.99	0.99	1.00	1.00	1.00	1.00	1.00	1.00	
10	T	RF	p	1.00	1.00	0.97	0.93	0.98	1.00	0.99	1.00	1.00	0.99	0.98
			r	0.99	0.99	0.95	0.97	0.97	0.99	1.00	0.99	0.98	1.00	
			f1	0.99	0.99	0.96	0.94	0.97	0.99	0.99	1.00	0.99	1.00	
10	F	*k*-NN	p	0.90	0.99	0.88	0.78	0.93	0.87	0.98	0.99	0.93	1.00	0.9
			r	0.92	0.76	0.84	0.88	0.66	1.00	0.99	0.98	0.99	1.00	
			f1	0.90	0.84	0.85	0.81	0.74	0.93	0.99	0.98	0.95	1.00	
10	F	SVM	p	0.97	0.99	0.95	0.94	0.98	0.97	1.00	0.99	0.99	1.00	0.97
			r	0.97	0.96	0.97	0.94	0.89	1.00	0.99	0.99	0.99	1.00	
			f1	0.96	0.97	0.96	0.93	0.93	0.98	1.00	0.99	0.99	1.00	
10	F	RF	p	0.97	0.94	0.92	0.95	0.91	0.87	1.00	0.99	0.97	0.98	0.94
			r	0.88	0.96	0.99	0.93	0.61	1.00	0.98	0.97	0.99	1.00	
			f1	0.90	0.94	0.95	0.94	0.69	0.92	0.99	0.98	0.98	0.99	
10	T-F	*k*-NN	p	1.00	1.00	0.98	0.97	1.00	0.98	1.00	0.99	0.99	1.00	0.99
			r	0.99	0.97	0.98	0.98	0.99	1.00	0.99	1.00	1.00	1.00	
			f1	1.00	0.98	0.98	0.98	0.99	0.99	0.99	1.00	0.99	1.00	
10	T-F	SVM	p	0.99	0.99	0.98	1.00	1.00	1.00	0.99	1.00	0.99	1.00	0.99
			r	0.99	0.99	0.99	0.98	0.99	1.00	0.99	1.00	0.99	1.00	
			f1	0.99	0.99	0.99	0.99	1.00	1.00	0.99	1.00	0.99	1.00	
10	T-F	RF	p	0.98	1.00	0.98	0.97	0.98	0.99	1.00	1.00	1.00	1.00	0.99
			r	0.98	0.98	0.98	0.98	0.98	1.00	1.00	1.00	0.99	1.00	
			f1	0.98	0.99	0.97	0.97	0.98	1.00	1.00	1.00	1.00	1.00	

### 3.2 Inter-subject classification performance

Table 5 shows the average classification results for the 5 repetitions. Better classification performance is observed for the 10 second windows as compared to the 4 second. Highest average accuracy’s of 91% and 97% are achieved with the time-frequency features in conjunction with *k*-NN or SVM for the 4- and 10-second windows, respectively. For both window lengths we observe that the combination of the SVM with any feature set and window length result in higher / equal accuracy as compared to the other classifiers.

**Table 5:** Inter-subject classification performance. Abbreviations: WL: window length, T: time, F: frequency EP: electrical panel, H: hoisting, Ld: ladder, Lf: lifting; OH: overhead; P: pushing; St: sitting; Sd: standing; Tp: typing; W: walking.

WL (s)	Feature	Model	Task-Specific Performance in the Test Set											Test Acc (stdev)
				EP	H	Ld	Lf	OH	P	St	Sd	Tp	W	
4	T	*k*-NN	f1	0.84	0.81	0.7	0.79	0.97	0.87	0.83	0.84	0.82	0.94	0.83 (0.03)
			p	0.81	0.85	0.72	0.75	0.99	0.88	0.84	0.86	0.81	0.95	
			r	0.88	0.78	0.7	0.85	0.94	0.86	0.83	0.83	0.83	0.94	
4	T	SVM	f1	0.87	0.83	0.77	0.83	0.98	0.88	0.9	0.87	0.88	0.95	0.86 (0.04)
			p	0.84	0.84	0.75	0.84	0.99	0.9	0.87	0.86	0.93	0.98	
			r	0.9	0.82	0.8	0.81	0.97	0.85	0.94	0.89	0.84	0.93	
4	T	RF	f1	0.85	0.8	0.74	0.8	0.97	0.87	0.88	0.85	0.87	0.95	0.84 (0.03)
			p	0.83	0.81	0.7	0.83	0.98	0.89	0.89	0.89	0.91	0.97	
			r	0.87	0.8	0.79	0.78	0.96	0.85	0.88	0.83	0.84	0.94	
4	F	*k*-NN	f1	0.84	0.76	0.72	0.76	0.78	0.88	0.91	0.91	0.9	0.98	0.83 (0.02)
			p	0.84	0.93	0.67	0.72	0.95	0.85	0.88	0.88	0.92	1	
			r	0.83	0.65	0.78	0.8	0.66	0.92	0.94	0.94	0.9	0.97	
4	F	SVM	f1	0.88	0.82	0.78	0.83	0.89	0.92	0.91	0.9	0.92	0.98	0.87 (0.02)
			p	0.87	0.83	0.76	0.87	0.91	0.93	0.89	0.87	0.93	0.99	
			r	0.89	0.82	0.79	0.8	0.86	0.91	0.94	0.94	0.9	0.97	
4	F	RF	f1	0.85	0.8	0.74	0.81	0.84	0.87	0.93	0.91	0.91	0.98	0.85 (0.02)
			p	0.81	0.86	0.74	0.81	0.9	0.86	0.94	0.9	0.89	0.96	
			r	0.88	0.76	0.75	0.82	0.79	0.88	0.92	0.91	0.94	0.99	
4	T-F	*k*-NN	f1	0.93	0.9	0.82	0.85	0.97	0.94	0.93	0.89	0.93	0.99	0.91 (0.02)
			p	0.9	0.95	0.84	0.82	0.99	0.94	0.92	0.86	0.95	0.99	
			r	0.96	0.87	0.81	0.87	0.95	0.94	0.95	0.93	0.93	0.99	
4	T-F	SVM	f1	0.94	0.91	0.84	0.87	0.97	0.95	0.94	0.89	0.94	0.97	0.91 (0.02)
			p	0.93	0.9	0.83	0.89	0.98	0.97	0.94	0.87	0.95	0.99	
			r	0.95	0.92	0.85	0.85	0.97	0.93	0.94	0.92	0.94	0.96	
4	T-F	RF	f1	0.9	0.87	0.81	0.84	0.97	0.93	0.93	0.89	0.94	0.98	0.89 (0.03)
			p	0.89	0.9	0.77	0.87	0.97	0.95	0.95	0.9	0.96	0.98	
			r	0.91	0.85	0.86	0.82	0.97	0.91	0.91	0.88	0.93	0.97	
10	T	*k*-NN	f1	0.85	0.89	0.82	0.82	0.98	0.91	0.84	0.98	0.82	0.96	0.88 (0.04)
			p	0.82	0.91	0.85	0.78	0.99	0.92	0.86	0.98	0.82	0.98	
			r	0.89	0.87	0.8	0.88	0.98	0.9	0.83	0.98	0.83	0.94	
10	T	SVM	f1	0.84	0.92	0.88	0.87	0.99	0.9	0.91	0.98	0.89	0.97	0.91 (0.04)
			p	0.77	0.93	0.88	0.87	0.99	0.95	0.88	0.98	0.94	1	
			r	0.92	0.91	0.88	0.87	0.99	0.86	0.94	0.99	0.85	0.95	
10	T	RF	f1	0.87	0.87	0.85	0.83	0.98	0.93	0.93	0.98	0.92	0.99	0.9 (0.04)
			p	0.84	0.87	0.84	0.87	0.98	0.94	0.94	0.97	0.92	0.99	
			r	0.9	0.88	0.86	0.81	0.98	0.92	0.92	0.99	0.92	0.99	
10	F	*k*-NN	f1	0.87	0.84	0.85	0.82	0.8	0.95	0.92	0.97	0.9	0.98	0.89 (0.02)
			p	0.86	0.98	0.8	0.82	0.97	0.92	0.87	0.94	0.9	1	
			r	0.89	0.75	0.92	0.82	0.68	0.98	0.97	0.99	0.9	0.97	
10	F	SVM	f1	0.91	0.91	0.93	0.94	0.92	0.99	0.96	0.99	0.95	0.99	0.95 (0.01)
			p	0.91	0.87	0.92	0.96	0.95	0.99	0.95	0.98	0.98	1	
			r	0.92	0.94	0.94	0.92	0.9	0.98	0.98	0.99	0.93	0.98	
10	F	RF	f1	0.89	0.92	0.91	0.92	0.9	0.95	0.94	0.98	0.95	0.99	0.93 (0.01)
			p	0.89	0.92	0.89	0.94	0.96	0.92	0.97	0.99	0.97	0.98	
			r	0.9	0.93	0.94	0.91	0.85	0.97	0.92	0.98	0.94	1	
10	T-F	*k*-NN	f1	0.97	0.96	0.95	0.93	0.99	0.99	0.96	0.99	0.95	1	0.97 (0.01)
			p	0.97	0.99	0.94	0.92	1	0.99	0.94	0.98	0.96	1	
			r	0.98	0.93	0.96	0.95	0.98	0.99	0.97	0.99	0.94	1	
10	T-F	SVM	f1	0.97	0.94	0.96	0.95	0.99	1	0.96	0.98	0.97	0.99	0.97 (0.01)
			p	0.97	0.94	0.96	0.93	0.99	1	0.95	0.99	0.99	1	
			r	0.97	0.95	0.97	0.96	0.98	0.99	0.97	0.98	0.96	0.97	
10	T-F	RF	f1	0.94	0.95	0.95	0.94	0.98	0.96	0.97	0.99	0.96	0.99	0.96 (0.01)
			p	0.93	0.96	0.93	0.96	0.98	0.97	0.97	0.98	0.98	1	
			r	0.95	0.94	0.97	0.93	0.98	0.95	0.98	0.99	0.94	0.99	

**Fig 5:**
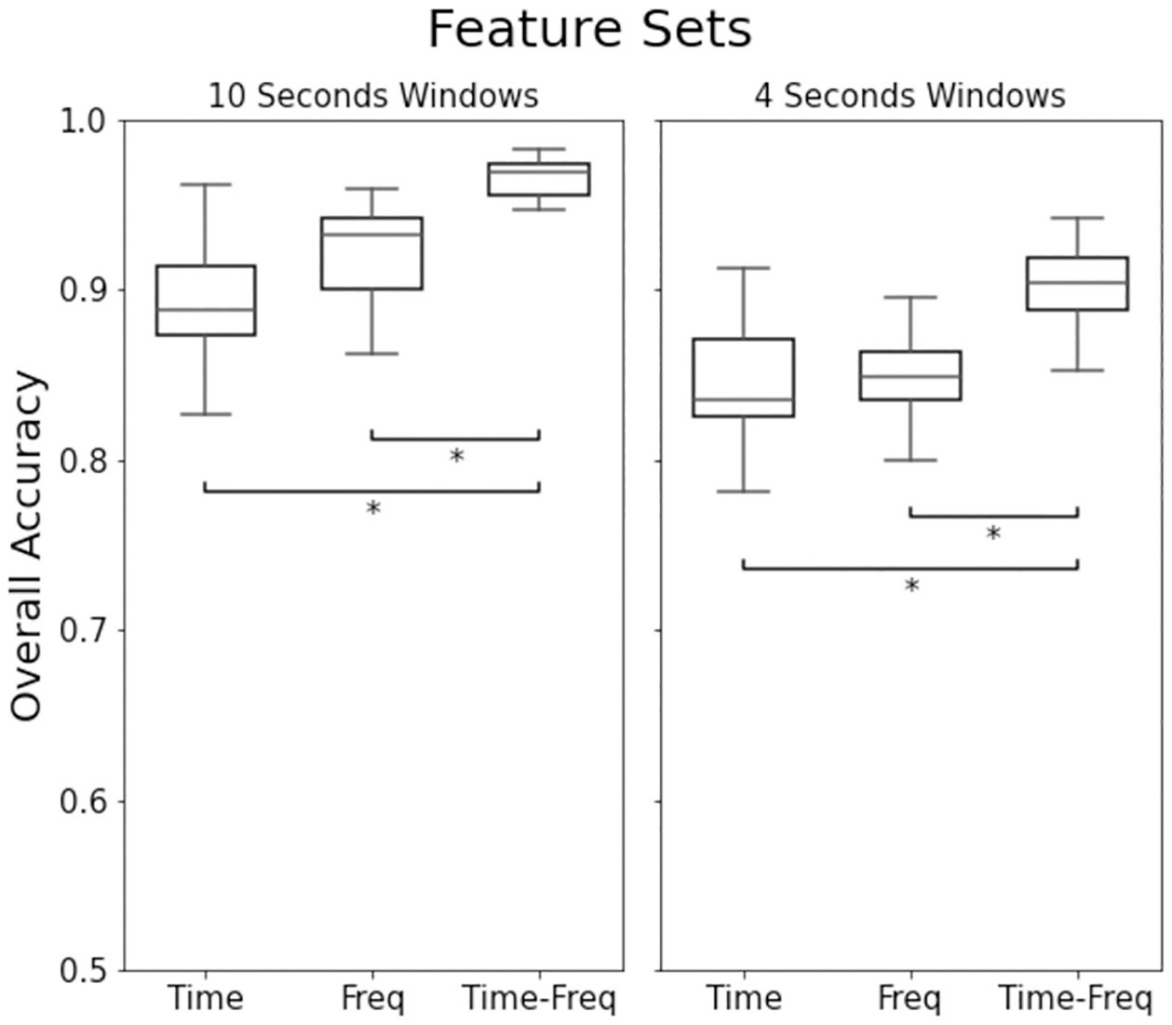
Inter-subject overall accuracy’s for the feature sets, presented separately for each window length. The * indicates statistically significant difference (p < 0.05) between pairs.

For both window lengths, the results of the ANOVA test showed significant main effects of feature set and classifier on model performance, with no significant interaction effect (Table 6). Post hoc analysis showed significant differences between the performance of the time-frequency domain features and both of the separate time domain and frequency domain feature sets. There were no significant pairwise differences between the classifiers (Table 6). We present the overall accuracies of each feature set in Fig 5 using separate box plots for the two window lengths.

**Table 6:** Two-way ANOVA for main and interaction effects of classifier and feature on inter-subject overall accuracy and Tukey’s HSD for significant effects (p < 0.05). Analyses are performed separately for each window length.

WL (sec)	Factors	ANOVA		Tukey’s HSD	
		*p* value		Factor Pairs	*p* value
4	Classifiers	0.015	0.485 (interaction)	*k*-NN vs RF	0.778
				*k*-NN vs SVM	0.111
				SVM vs RF	0.346
	Features	0.000		Time vs Freq	0.864
				Time vs Time-Freq	0.001
				Freq vs Time-Freq	0.001
10	Classifiers	0.005	0.128 (interaction)	*k*-NN vs RF	0.249
				*k*-NN vs SVM	0.086
				SVM vs RF	0.824
	Features	0.000		Time vs Freq	0.060
				Time vs Time-Freq	0.001
				Freq vs Time-Freq	0.001

### 3.3 Overall feature importance

**Fig 6:**
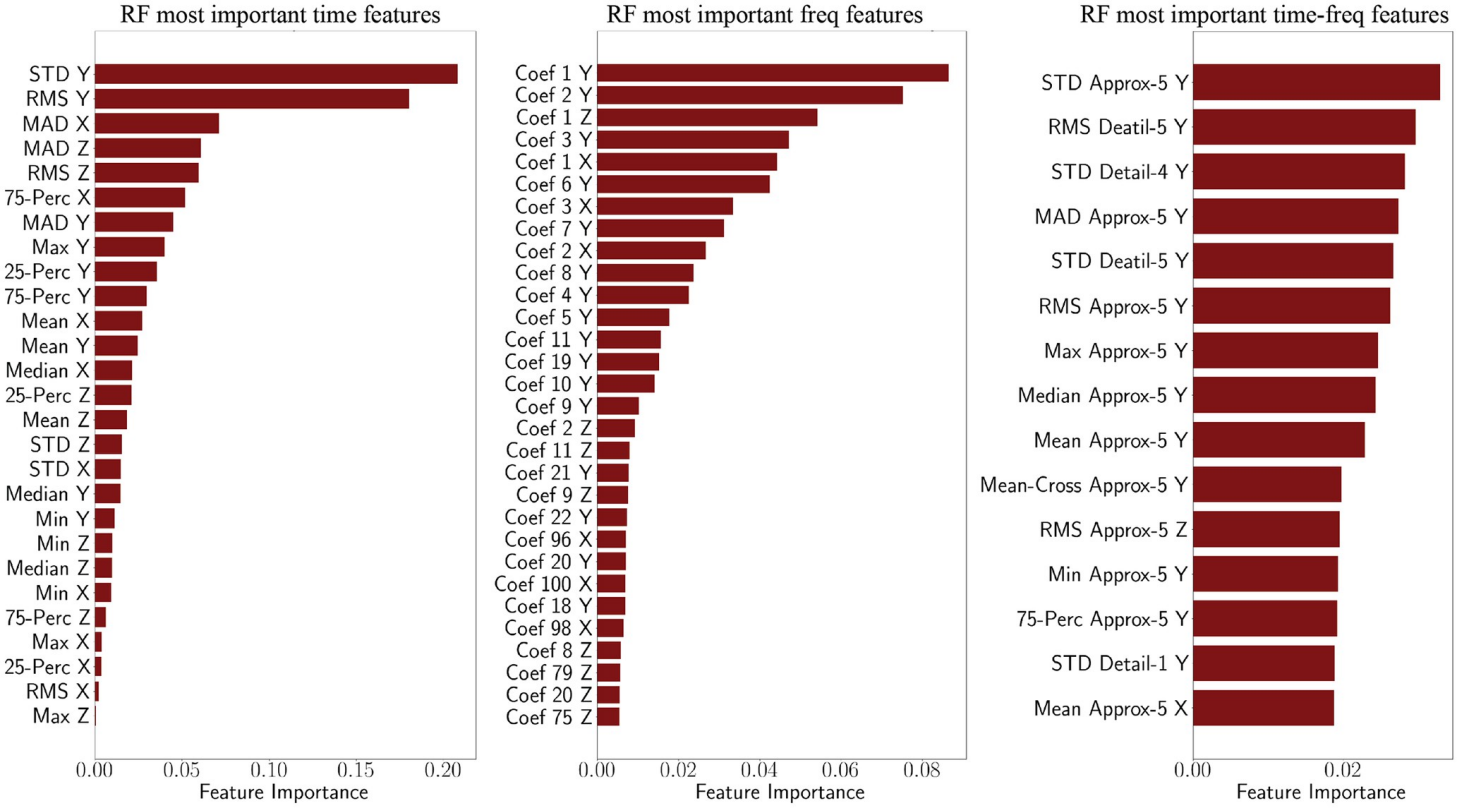
Overall feature importance from RF.

In Fig 6 we present the most important features, along with their contributions, obtained from the RF classifier for the case of the 10 second windows. For the time domain, since only 27 features were used, the importance of all features are presented. However, for the frequency and time-frequency domain sets, given the large number of features the top 30 and 15 important features, respectively, are included. An important observation from the importance plots is that the most important features are from the *Y* acceleration signal, implying that the *Y* acceleration is, in general, more dissimilar across different tasks, and therefore more distinctive. While this is an informative observation, the importance plots do not provide further details about the contribution of each feature for individual activities.

### 3.4 Class-specific feature importance using the LIME method

We show the results for the methods mentioned in Section 2.6 here, focusing on the features from the frequency and time-frequency domains. As an illustration, we identified the important features for prediction of two activities, i.e., working at an electrical panel and hoisting a weighted bucket. For the case of the 10 second windows, we identified the 30 most important frequency features and used the inverse Fourier transform to reconstruct the three acceleration signals. For time-frequency domain (wavelet) features, after finding the important features, we used the coefficients from which the statistics were derived to reconstruct the signal using the inverse wavelet transform. Therefore, we used the 15 most important features for the reconstruction. Since the LIME method is model agnostic, we performed the analysis with only one classifier, i.e., *k*-NN. Moreover, since LIME is a local interpretation approach, we applied the method on 30 observations that were correctly classified for the two tasks. The feature contributions were averaged over the 30 observations.

#### 3.4.1 Electrical panel

**Fig 7:**
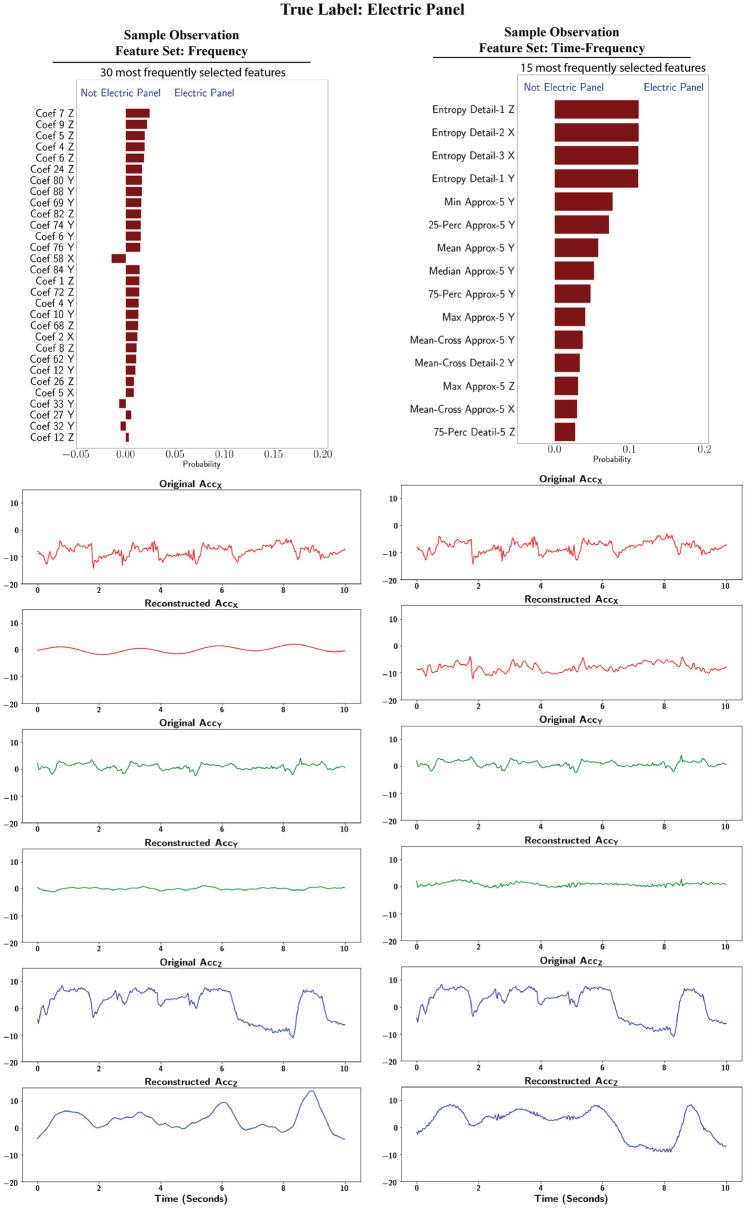
LIME interpretation: Class specific feature importance and reconstruction for electric panel.

The top panel in Fig 7 presents the feature importance for the electrical panel task from LIME method for the frequency and time-frequency features. From this figure, the features from *Z* acceleration are the most important in both feature sets. Also, from the time-frequency domain features, the second and third most important features are selected from the *X* acceleration signals. These observations are in contrast with the overall feature importance from the RF classifier, and reaffirm that feature importance should be investigated for individual classes. We selected a sample observation out of the 30 observations used to calculate the feature importance for electrical panel and reconstructed the signal using the important features. The reconstructions from the frequency and time-frequency features are presented in the bottom panels of Fig 7.

For the frequency domain features, only two (out of 30) were selected from the *X* signal. The reconstruction shows a simple periodic pattern. From the time-frequency domain features, three (out of 15) features were selected from the *X* signal and the reconstruction from the time-frequency domain features set showed an emphasis on the abrupt changes from the *X* signal to classify the task.

The important frequency features for the *Y* signal were mainly selected from higher frequencies and therefore the reconstruction does not capture the dominant pattern in the signal. This could be due to similar dominant pattern in the *Y* signal among different tasks and more variation in the *Y* signal for the electrical panel. However, the majority of important time-frequency domain features were selected from the last approximation coefficient. As can be seen in the figure, this helped capture the 1^*st*^ two peaks that were present in the signal, and the more steady pattern present towards the end. Moreover, two other features were selected from the 1^*st*^ two detail coefficients of the *Y* signal, which appear as more noise in the reconstruction.

Comparing the *Z* acceleration signal against the other two highlights the dominance of the *Z* acceleration signal for this task. In frequency domain, the first six important features were from the *Z* acceleration. In the time-frequency domain, the most important feature was also selected from the *Z* axis.

#### 3.4.2 Hoisting

**Fig 8:**
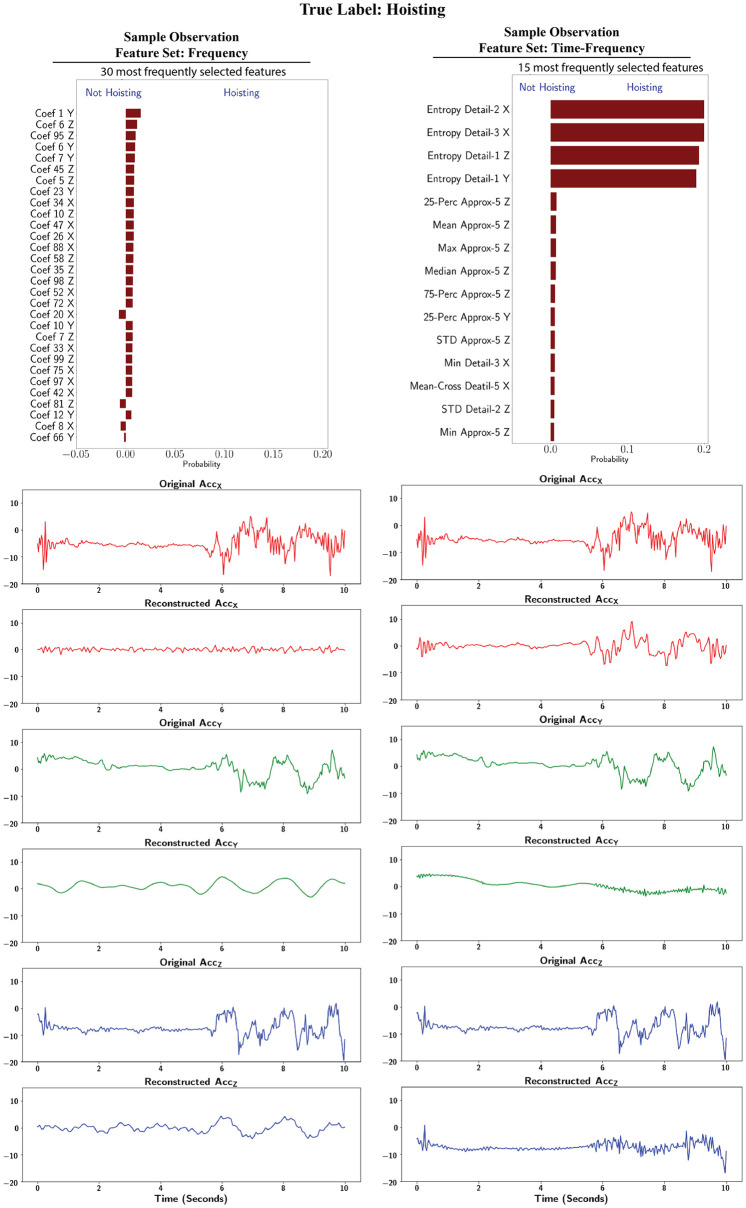
LIME interpretation: Class specific feature importance and reconstruction for Hoisting.

Fig 8 presents the LIME feature importance and reconstruction results for the hoisting task. From the original signals in the bottom panels it can be observed that, unlike the electrical panel, there was no one acceleration component that dominated in terms of magnitude or pattern.

Frequency features from all three acceleration signals were among the most important features, with similar levels of contribution. However, the majority of the selected features from the *X* signal were from frequencies higher than 3 Hz The reconstructions show that for the *X* signal, the high frequency values were selected as the distinctive characteristics of the activity. Comparing the original *Y* and *Z* signals against the *X* signal, it is evident that the *X* acceleration values include larger variation, and this can be a valid distinctive factor between hoisting and other activities.

For the time-frequency domain, the four most important features had higher contributions compared to the remaining selected features. Only two features from the *Y* acceleration were selected, and this is reflected in the reconstructions for the *X* and *Z* signals, where the shapes of their original counterparts were captured more accurately.

## 4 Discussion and conclusions

### 4.1 Summary of the main contributions

In this paper we examined the utility of a single accelerometer, worn on the dominant wrist, for the classification of common work activities among ELWs. We evaluated three main research questions that pertain to (1) the utility/accuracy of individualized models for classifying ELW tasks, (2) the feasibility of constructing a generalized model that does not need to be tuned for new workers, and (3) communicating the relative importance and impact of our proposed/engineered features on the performance of the classification models. An experimental lab study was designed to assess the research questions, where 37 participants were recruited to perform 10 simulated ELW tasks.

Based on our proposed modeling approach and the experimental study, this paper makes five main contributions. First, we have shown that the features captured from a single wrist-worn sensor can be used to correctly classify complex and dynamic ELW tasks. Second, our classification accuracies were similar for both the individualized and generalized models. This is a practically significant finding since it presents researchers/practitioners with flexibility to train models based on a limited number of workers without the need to have ELWs perform all tasks (i.e., the ELWs’ data can be used even if they do not complete all tasks as long as they are expected to follow the prescribed work instructions). Third, the feature importance and the LIME method indicate that certain tasks show a dominant signal in one of the three Cartesian coordinates. While this can be expected from a biomechanics perspective, our ability to show and quantify these effects is important in providing justification of the suitability of our engineered features and communicating model findings in a “clear box” manner. Fourth, we showed that 10-second windows present slightly higher classification accuracies; however, their classification performance is not substantially better than the shorter 4-second window (which is the minimum time unit to guarantee a full task cycle based on our most complex task). The implication is that near-real time models for task classification can be deployed. Fifth, time-frequency features resulted in a relatively stable testing accuracy across the intra- and inter-subject deployment scenarios for three investigated machine learning models.

### 4.2 Relevance to human performance monitoring research/ practice

It is important to contextualize our work in the context of the existing human activity recognition (HAR) literature. The results from our experimental study advances the HAR literature in two major ways. First, our inter-subject modeling scenario is more conservative and complex than the studies highlighted in Table 1 since we (1) did not include data for any of the test subjects in our training set; and (2) had a relatively large number of tasks, 10, when compared to those studies (ranging from 3 to 11 with a median of 5). Hence, our reported accuracies of ≥ 91% for the 4-second window, which increases to ≥ 97% with 10-second windows, is excellent when compared to those studies where the accuracy ranges from 66.8% to 98.0% with a median of 90.1%. Second, we captured how features engineered from the sensor contribute to an obtained classification result. The acceleration in certain activities showed a dominant signal in one of the three Cartesian coordinates. In some cases this is due to a unique movement that associates with the relevant task and does not appear in other activities (e.g., the *Z* acceleration signal was the dominant signal for classifying “electrical panel” task). Moreover, if higher dispersion is a characteristic of certain activities, the features are expected to be selected from higher frequencies for that activity. The hoisting, as one of the tasks that induced the largest load on the upper body, caused some degree of quivering. As expected, the signal reconstructions for the hoisting task resulted in more noisy signals.

### 4.3 Limitations and suggestions for future research

There are three limitations that may impact the generalizability of our results. First, a laboratory experiment is a tightly controlled simulation of the real workplace and lacks the heterogeneities inherent in a field study. Our work was based on 37 subjects and this large number of subjects was unprecedented in previous studies. Although this can mitigate the heterogeneity issue, a field study can be better generalized to other cases. Second, our study was designed such that the repetitive tasks were performed for ten or twenty repetitions and the time-based tasks continued for three minutes. However, in the real world applications different tasks can be performed intermittently and for fewer repetitions / shorter duration. This scenario, which we call a mixed task scenario, is more challenging in terms of annotation and windowing, and may require automatic change point detection for creating the windows. Third, we did not study the impact of muscle fatigue on the performance of the proposed activity recognition framework, while fatigue presence may cause changes in the pattern of later repetitions of each task or the tasks that are performed near to the end of simulation.

Finally, we provide three recommendations for future research. First, studies should investigate how active learning can complement our framework. While our current classification accuracy is excellent for both intra-subject and inter-subject scenario, it will suffer from a limited number of training labels. To alleviate this and maintain a good accuracy, one can use active learning, where the learning algorithm is allowed to query a subject to label the task that he/she is performing. Meanwhile, a penalty for the request label action should be considered, because excessive queries can complicate the performance of the subjects. Also, as mentioned above, change point detection is a prerequisite for the mixed task scenario. Towards this end, future research should be devoted to the development of a real-time segmentation approach for detecting the task transition times. Moreover, the data between consecutive change points can be further segmented into cycles for repetitive tasks. These segments can be used to investigate the effect of fatigue on the patterns of task performance. Finally, the real-time implementation of activity recognition is of great importance in industrial applications for worker monitoring purposes. We suggest that the future research should investigate and address the challenges in real-time implementation of the proposed algorithms.

## Supporting information

S1 File (BST)
